# Complex Aortic Graft Infection with Giant Collection after Bentall-Bono Surgery: Diagnostic and Therapeutic Challenges

**DOI:** 10.1016/j.jaccas.2025.106723

**Published:** 2026-01-28

**Authors:** Fátima Sol Cabrera, Pau Rello Sabate, Filipa Xavier Valente, Nuria Fernandez-Hidalgo, Nuria Vallejo Camazón, Albert Roque, Gisela Teixidó-Taura, Jose F. Rodriguez-Palomares

**Affiliations:** a Department of Cardiac Imaging, Hospital Universitari Vall d’Hebron, Vall d’Hebron Institut de Recerca (VHIR), Vall d’Hebron Barcelona Campus Hospitalari, Barcelona, Spain; b Departament de Medicina, Universitat Autònoma de Barcelona, Barcelona, Spain; c Servei de Cardiologia Clínica, Hospital Universitari Vall d’Hebron, Barcelona, Spain; d Servei de Malalties Infeccioses, Hospital Universitari Vall d’Hebron, Vall d’Hebron Institut de Recerca (VHIR), Vall d’Hebron Barcelona Campus Hospitalari, Barcelona, Spain; e CIBERINFEC, ISCIII-CIBER de Enfermedades Infecciosas, Instituto de Salud Carlos III, Madrid, Spain; f Department of Radiology, Hospital Universitari Vall d’Hebron, Barcelona, Spain; g Department of Nuclear Medicine, Hospital Universitari Vall d’Hebron, Barcelona, Spain; h Cardiovascular Imaging Section and Aortic Diseases Unit (VASCERN), Department of Cardiology, Vall d’Hebron Hospital Universitari, Vall d’Hebron Barcelona Hospital Campus, Vall d'Hebron Institut de Recerca (VHIR), Barcelona, Spain; i Centro de Investigación Biomédica en Red de Enfermedades Cardiovasculares (CIBERCV), Instituto de Salud Carlos III, Madrid, Spain

**Keywords:** aorta, blood tests, chest pain, complication, computed tomography, dissection, echocardiography, endocarditis, postoperative, valve replacement

## Abstract

**Background:**

Aortic graft infection (AGI) is a rare but life-threatening complication after aortic root surgery, particularly when postoperative collections are present.

**Case Summary:**

A 49-year-old man developed progressive perigraft enlargement followed by fever and chest pain 8 months after Bentall-Bono surgery. He had recently undergone a dental procedure without antibiotic prophylaxis. Blood cultures grew *Enterococcus faecalis*, and transesophageal echocardiography revealed a heterogeneous perigraft collection with communication to the aortic lumen, as well as graft vegetations and partial suture dehiscence. These findings fulfilled the Management of Aortic Graft Infection Collaboration criteria for AGI. The patient underwent urgent graft replacement using a hemi-Cabrol technique, with an uncomplicated postoperative course.

**Discussion:**

This case illustrates the diagnostic complexity of AGI in patients with altered postsurgical anatomy, where distinguishing infection from chronic collections is challenging. It highlights the pivotal role of multimodality imaging and coordinated multidisciplinary management.

**Take-Home Message:**

Early recognition and timely surgical intervention are essential to improving outcomes in AGI.

## Graphical Abstract

**Figure:**
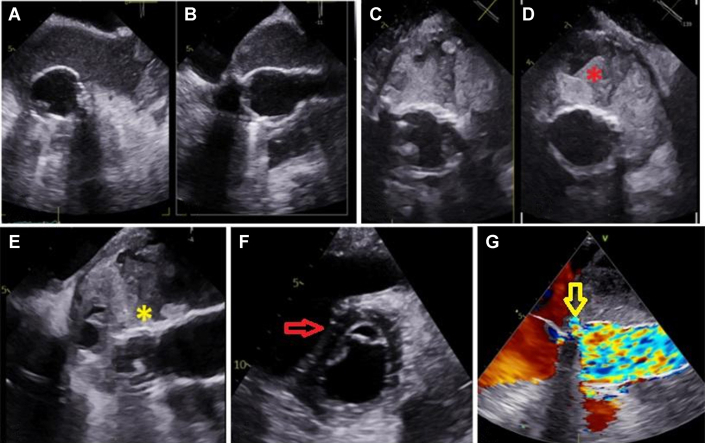

## History of Presentation

A 49-year-old man with hypertension, dyslipidemia, right bundle branch block, and a history of type A aortic dissection treated with Bentall-Bono surgery presented with recurrent chest pain and fever. Postoperatively, a perigraft collection was noted on computed tomography (CT) but was managed conservatively given the patient's stable condition and lack of leak on transesophageal echocardiography (TEE). Six months later, he suffered a hemorrhagic stroke. Two months after the stroke, he presented with intermittent chest pain recurring over a period of 3 months, accompanied by a 1-week history of fever and vegetative symptoms. The patient reported a recent lost dental crown without antibiotic treatment. On admission, he was conscious and oriented, with a temperature of 39.5 °C, a blood pressure of 135/83 mm Hg, a heart rate of 110 beats/min, and mild dyspnea (oxygen saturation: 95%). Cardiovascular examination revealed prosthetic clicks and no murmurs.

## Past Medical History

- Arterial hypertension
- Dyslipidemia
- Complete right bundle branch block
- Underwent type A aortic dissection repair with Bentall-Bono surgery in November 2023 (mechanical prosthesis)
- Hemorrhagic left hemispheric stroke in May 2024 (2 months before current presentation)
- Family history: maternal uncle with abdominal aortic surgery at age 50; no known genetic aortopathies.

## Differential Diagnosis

Our initial considerations spanned a spectrum of possibilities, each demanding careful exclusion.

- Prosthetic valve endocarditis with aortic graft infection (AGI)
- Sterile perigraft collection with inflammatory response
- Aortic pseudoaneurysm with superimposed infection
- Recurrent aortic dissection or leak
- Noncardiac source of fever and chest pain

## Investigations

**Figure 1:**
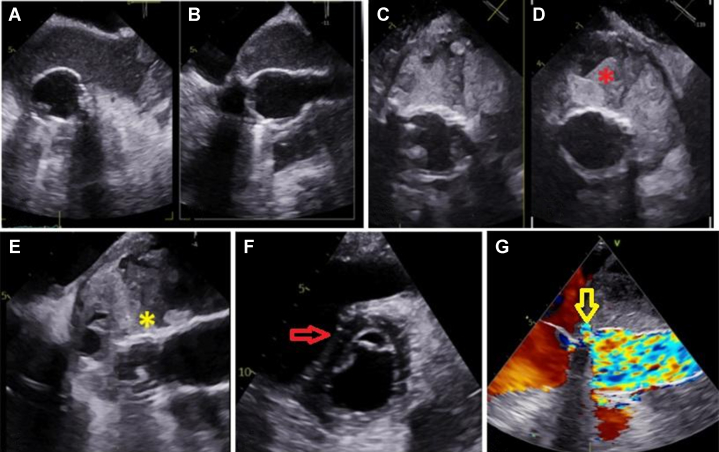
Findings on Transesophageal Echocardiography Transesophageal echocardiography demonstrating a large perigraft fluid collection surrounding the aortic conduit (A and B), with heterogeneous and thickened internal material compatible with abscess and debris (C and D). Mobile echodensity suggestive of vegetation (yellow asterisk) attached to the prosthetic aortic graft (E). (D) Red asterisk indicates the periprosthetic abscess adjacent to the aortic graft. (E) Yellow asterisk indicates the prosthetic aortic conduit and vegetation attached to the aortic graft. Marked diastolic interventricular septal flattening (red arrow) indicating elevated right-sided pressures (F). A focal jet on color Doppler (yellow arrow) demonstrates flow between the perigraft cavity and the proximal Bentall anastomosis line (G).
- Blood cultures: positive for *Enterococcus faecalis* (all bottles from 4 sets of blood cultures).
- Urgent CT angiography: rapid expansion of the perigraft collection with compression of adjacent structures (Figure 2).

- Laboratory results: elevated C-reactive protein (3.40 mg/dL), erythrocyte sedimentation rate (96 mm/h), anemia (hemoglobin: 11.2 g/dL), elevated N-terminal pro–B-type natriuretic peptide (2308 pg/mL), troponin I (23 ng/mL), estimated glomerular filtration rate (>90 mL/min/1.73 m^2^), creatinine (0.78 mg/dL), urea (20.0 mg/dL).
- Electrocardiogram: sinus rhythm with complete right bundle branch block and signs of left ventricular hypertrophy.
- Chest x-ray: mediastinal widening.
- Cardiac CT: enlargement of perigraft collection with pseudoaneurysm.
- TEE: heterogeneous perigraft collection communicating with the aortic lumen at the proximal Bentall suture, mobile hyperechogenic image on the vascular graft suggestive of vegetation (Video 1), residual type B dissection, preserved left ventricular function, diastolic septal flattening, moderate tricuspid regurgitation (pulmonary arterial pressure: 60 mm Hg) (Figure 1).

**Figure 2:**
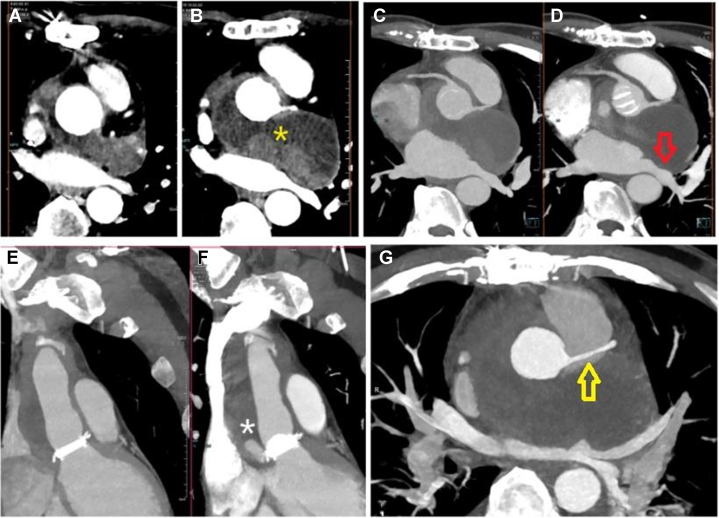
Findings on Computed Tomography Contrast-enhanced cardiac CT showing marked and rapid expansion of the perigraft fluid collection surrounding the ascending aortic conduit (A and B). Multiplanar reconstructions demonstrate focal dehiscence at the proximal suture line of the valved conduit with contrast extension toward the perigraft space, consistent with an anastomotic leak (E and F). The enlarging collection exerts mass effect on adjacent structures, producing extrinsic compression of the left superior pulmonary vein (red arrow) followed by significant narrowing of the right pulmonary artery (yellow arrow) (C, D, and G). (B) The yellow asterisk denotes the perigraft fluid collection. (F) The white asterisk denotes the focal dehiscence at the proximal suture line of the valved conduit. CT = computed tomography.

## Management

Medical management consisted of empirical antibiotic therapy with intravenous ampicillin 2 g/4 h plus ceftriaxone 2 g/12 h, which was initiated immediately upon the positive blood culture result (July 7, 2024). The case was urgently evaluated by a multidisciplinary team comprising cardiologists, cardiac surgeons, infectious disease specialists, and imaging experts. The team reached a consensus to proceed with surgical reintervention (July 10, 2024). However, the patient experienced rapid clinical deterioration, with hypotension and chest pain, and imaging demonstrated rapid expansion of the perigraft collection; therefore, emergency surgery was performed.

**Figure 3:**
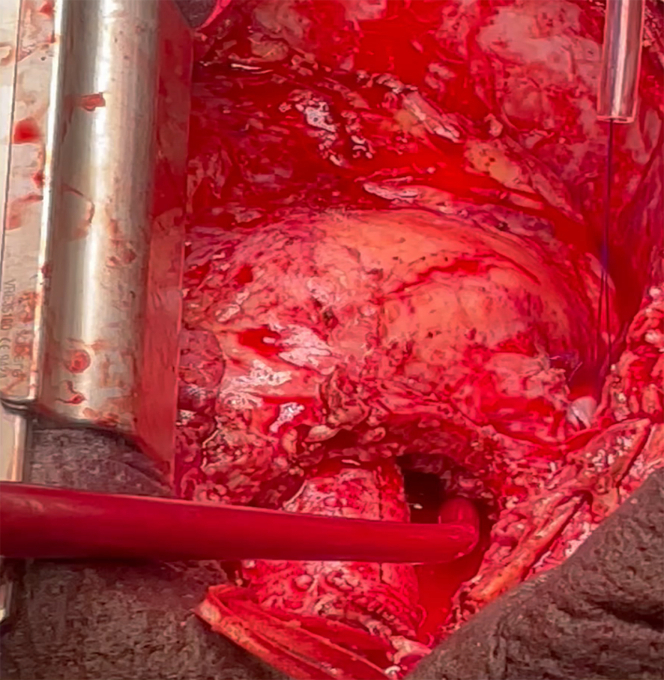
Intraoperative Findings Revealing a Large Perigraft Cavity Containing a Mixture of Blood and Thick Purulent Material, Which Drained Immediately Upon Surgical Exposure A clear dehiscence of the proximal suture line of the valved conduit was identified, corresponding to the site of communication with the infected perigraft collection.

During surgical intervention (July 14, 2024), a large peritube collection filled with blood and purulent material was obtained. Dehiscence of approximately half the circumference of the Hemashield vascular graft at the noncoronary and left coronary sinus levels was observed (Video 2, Figure 3). Complete graft explantation and replacement were performed with a 21/22-mm On-X conduit (Artivion, Inc), using a hemi-Cabrol technique (common trunk reconstruction). The material obtained during the surgical intervention was cultured, and all cultures (including the valve and the perigraft collection) were positive for *E* *faecalis*.

## Follow-Up

**Figure 4:**
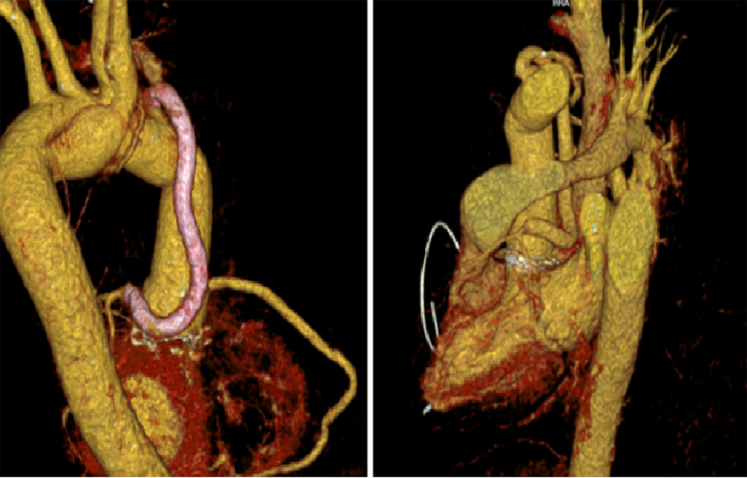
Three-Dimensional Cardiac CT Reconstruction Demonstrating Correct Positioning of a New Vascular Graft With a Mechanical Aortic Valve A hemi-Cabrol procedure was performed, consisting of a bypass conduit originating from the distal portion of the aortic prosthesis and anastomosed to the left main coronary trunk (highlighted in pink) ensuring unobstructed coronary perfusion. CT = computed tomography.

Postoperatively, the patient was admitted to the coronary care unit, requiring vasoactive support and electrical cardioversion for atrial fibrillation. He completed a course of intravenous ceftriaxone and ampicillin. A follow-up CT scan demonstrated a significant reduction in the perigraft collection size and a stable descending aorta size (Figure 4). The patient showed significant clinical improvement and was discharged home.

## Discussion

AGI lacks a universally accepted case definition, complicating its clinical approach. The MAGIC (Management of Aortic Graft Infection Collaboration) group proposed a multidisciplinary consensus-based definition, in which AGI is suspected in the presence of either 1 major criterion or ≥2 minor criteria from different categories: clinical/surgical, radiological, and laboratory.^1^ This framework facilitates diagnostic structuring in the context of a complex and heterogeneous clinical picture^1^ (Table 1).

**Table 1:** Diagnostic Criteria for Aortic Graft Infection

Clinical/Surgical	Radiology	Laboratory
• **Pus (confirmed by microscopy) around graft or in aneurysm sac at surgery** • **Open wound with exposed graft or communicating sinus** • **Fistula development (eg, aortoenteric or aortobronchial)** • **Graft insertion in an infected site (eg, fistula, mycotic aneurysm, or infected pseudoaneurysm)**	• **Perigraft fluid on CT scan ≥3 mo after insertion** • **Perigraft gas on CT scan ≥7 k after insertion** • **Increase in perigraft gas volume demonstrated on serial imaging**	• **Organisms recovered from an explanted graft** • **Organisms recovered from an intraoperative specimen** • **Organisms recovered from a percutaneous, radiologically guided aspirate of perigraft fluid**
• Localized clinical features of AGI (eg, erythema, warmth, swelling, purulent discharge, pain) • Fever ≥38 °C with AGI as most likely cause	• Other, for example, suspicious perigraft gas/fluid/soft tissue inflammation; aneurysm expansion; pseudoaneurysm formation; focal bowel wall thickening; discitis/osteomyelitis; suspicious metabolic activity on FDG PET/CT; radiolabeled leukocyte uptake	• Blood culture(s) positive and no apparent source except AGI • Abnormally elevated inflammatory markers with AGI as most likely cause (eg, ESR, CRP, WBC count)

AGI is diagnosed in the presence of a single major criterion (bolded text), plus any other criterion (major or minor) from another category: clinical/surgical, radiological, or laboratory. Table Adapted from Lyons OTA, Baguneid M, Barwick TD. Diagnosis of aortic graft infection: a case definition by the management of aortic graft infection collaboration (MAGIC) *Eur J Vasc Endovasc Surg*. 2016;52:758-763, with permission.

AGI = aortic graft infection; CT = computed tomography; CRP = C-reactive protein; ESR = erythrocyte sedimentation rate; FDG = fluorodeoxyglucose; PET = positron emission tomography; WBC = white blood cell.

The true incidence of AGI remains unknown, though it is estimated to range between 1% and 6% among patients with aortic grafts.^2^^,^^3^ It can occur at any time after surgery, and it is associated with high morbidity and mortality rates. Even in high-volume aortic centers, reported mortality often exceeds 20%.^4^^,^^5^ Risk factors include advanced age, prolonged surgical time, high American Society of Anesthesiologists classification, emergency surgery, and surgical site infection.^6^

Clinical presentation is highly variable, depending on the pathogen involved and the degree of systemic compromise. Symptoms are often nonspecific and necessitate further diagnostic testing. Computed tomography angiography (CTA) is a key diagnostic tool. Findings such as persistent fluid, gas formation, or anastomotic dehiscence are suggestive of AGI. Diagnostic accuracy is enhanced with ^18^F-fluorodeoxyglucose positron emission tomography (PET), alone or in combination with CTA, although standardized patterns and cutoff values remain undefined.^7^

AGI is classically divided into early (<4 months) and late (>4 months) infections. Early infections are typically caused by virulent organisms such as *Staphylococcus aureus* and present with abrupt onset, fever, and systemic toxicity. Late infections usually follow an indolent course and are often caused by less virulent skin flora. Definitive diagnosis requires microbiological confirmation from explanted grafts, percutaneous perigraft fluid aspiration, or appropriately processed autopsy specimens.^8^^,^^9^

Treatment is based on 2 pillars: surgical explantation of the infected graft and adjunctive antimicrobial therapy. However, graft explantation carries substantial surgical risk, with mortality ranging from 18% to 30%, whereas maintaining an infected prosthesis in situ can result in mortality approaching 100% within 2 years. Antibiotic therapy alone is reserved for inoperable patients. Surgery becomes emergent in the setting of imminent rupture or newly developed vascular discontinuity.^1^^,^^5^

This case presented several distinctive features. The perigraft collection was exceptionally large and appeared radiologically stable for months, masking its progressive infectious transformation. There was coexistence of aortic graft infection, pseudoaneurysmal changes, and prosthetic valve endocarditis. *E* *faecalis*—a less frequent pathogen in aortic graft infections—was identified in this late presentation. The patient experienced rapid clinical deterioration with abrupt enlargement of the collection, and surgical rescue was achieved despite the considerable operative complexity and hostile local tissue conditions.

The diagnosis fulfilled MAGIC criteria through multiple pathways. Clinically, the patient presented with fever and chest pain, and intraoperative inspection revealed graft dehiscence with purulent contents within the perigraft collection, meeting major surgical criteria. Radiologically, CTA demonstrated persistent perigraft fluid beyond 3 months, direct communication with the aortic lumen, and interval enlargement on serial imaging. Laboratory evaluation confirmed multiple positive blood cultures for *E* *faecalis*. PET/CT was not performed. Current consensus documents note that the optimal timing of PET/CT after aortic surgery is uncertain, as postoperative sterile inflammation can produce fluorodeoxyglucose uptake for several weeks, potentially mimicking infection. Guidelines advise caution when interpreting PET/CT studies within the first 2 to 3 postoperative months and recommend integrating uptake patterns with clinical and CT findings; focal, heterogeneous uptake tends to suggest infection, whereas linear and diffuse uptake is more consistent with sterile inflammation. Nuclear medicine guidelines also suggest that, in equivocal cases—particularly early after surgery—radiolabeled leukocyte scintigraphy may help differentiate between sterile inflammation and active infection.

The identification of *E* *faecalis* was clinically relevant. Although less common than *Staphylococcus* species, it is associated with subacute presentations, and the recent dental event without prophylaxis may represent a plausible source. Expert statements acknowledge that oral pathogens can seed cardiovascular prostheses in susceptible hosts.

The hemi-Cabrol technique was chosen because of infection and friability in the coronary button region, which precluded standard coronary reimplantation. This approach ensured a tension-free anastomosis in a contaminated field and allowed complete explantation of the infected conduit.

Surgical risk was high given active infection, urgent reoperation, and complex anatomy. The EuroSCORE II was 11.2%, and the Society of Thoracic Surgeons valve + aorta predicted mortality was 6.1%, both within the high-risk category. In addition, the patient met several features identified in Society for Vascular Surgery/Society of Thoracic Surgeons consensus statements as markers of high-risk aortic graft infection, such as purulent perigraft collections, graft dehiscence, systemic infection, and a reoperative field, further underscoring the severity of the presentation.

Multimodality imaging and multidisciplinary collaboration were essential to achieving a favorable outcome. The integration of CTA, TEE, clinical findings, and microbiological confirmation supported the need for early surgical intervention. This case illustrates how the structured application of the MAGIC criteria and the Duke–International Society criteria for prosthetic valve endocarditis,^10^ the repetition of imaging when new symptoms arise, and prompt surgical management can improve outcomes in a condition with intrinsically high mortality.

## Conclusions

This case demonstrates the complexity of diagnosing and managing AGI, a rare but severe condition with a potentially fatal course if not diagnosed and treated promptly. Its diagnosis is challenging, as symptoms are often nonspecific and depend on the causative pathogen. Treatment typically requires surgical intervention, since antibiotic therapy alone is usually insufficient. Therefore, a comprehensive multidisciplinary approach is essential in all cases.

Currently, there are no randomized clinical trials that robustly support therapeutic decisions, making case reports and expert consensus documents crucial for guiding clinical practice. The case presented highlights how a multidisciplinary team approach as well as prompt diagnosis and intervention can significantly improve the prognosis of this high-risk condition.

## Funding Support and Author Disclosures

The authors have reported that they have no relationships relevant to the contents of this paper to disclose.

## Appendix

Video 1 Transesophageal echocardiography showing aortic graft collection and vegetation.

Video 2 Giant mediastinal fluid collection surrounding the aortic graft.
